# Nanorobots and Exosomes: Driving the New Frontier for Bladder Tumors through Non-Coding RNAs

**DOI:** 10.32604/or.2026.078045

**Published:** 2026-09-14

**Authors:** Lorenzo Spirito, Cristina Quintavalle, Paola Coppola, Matteo Esposito, Francesco Esposito, Gabriella De Vita, Pierlorenzo Pallante

**Affiliations:** 1 Urology Unit, Department of Woman, Child and General and Specialized Surgery, University of Campania “Luigi Vanvitelli”, Naples, Italy; 2 Institute of Endotypes in Oncology, Metabolism and Immunology (IEOMI) “G. Salvatore”, National Research Council (CNR), Naples, Italy; 3 Department of Molecular Medicine and Medical Biotechnology (DMMBM), University of Naples “Federico II”, Naples, Italy

**Keywords:** Nanorobot, exosome, non-coding RNA, bladder cancer

## Abstract

Because of its limited treatment choices and high recurrence rates, bladder cancer (BCa) presents a significant clinical issue. As a result, current research is concentrated on creating novel approaches for early diagnosis and more specialized treatments. Nanorobots hold significant potential as precise medicine-delivery systems and as *in situ* diagnostic vectors within this dynamic environment. Personalized treatments are becoming increasingly feasible thanks to new insights into the molecular pathways driving tumor growth, revealed through studies on exosomes and non-coding RNAs (ncRNAs), however, their true potential lies in integrating these findings. This review analyzes the role of nanorobots, exosomes and ncRNAs in BCa and, more importantly, proposes a synergistic framework where active nanorobots are used for the targeted delivery of therapeutic exosomes (loaded with ncRNAs), overcoming the physiological limitations of intravesical administration. The interplay between nanorobots, exosomes and ncRNAs outlines a coherent and synergistic therapeutic approach with nanorobots enabling active navigation, exosomes offering stable and biocompatible loading, and ncRNAs providing the precision molecular functions required for targeted intervention. Their integration constitutes the core rationale driving this emerging approach and this technological convergence may open new avenues for precision diagnosis and targeted therapy. Incorporating these state-of-the-art technologies could ultimately transform the treatment of BCa and greatly enhance patient outcomes and quality of life. The aim of this review is to evaluate the current evidence on these three technologies and to define an integrated strategy that addresses existing clinical barriers and advances precision medicine in BCa.

## 1 Nanotechnology and Molecular Therapeutics for Current Challenges in Bladder Cancer

### 1.1 Current Issues in Bladder Cancer

Bladder cancer (BCa) remains a major global health burden, ranking among the most frequently diagnosed malignancies and contributing substantially to cancer-related mortality worldwide. Recent international assessments report approximately 614,000 new cases and about 220,000 deaths in 2022 [1], confirming its persistent epidemiological impact across regions. Global cancer surveillance analyses further demonstrate marked geographic variation in incidence and mortality, driven largely by differences in tobacco consumption, occupational and environmental exposure, demographic ageing, and metabolic conditions, with higher burdens observed in several income countries and in rapidly transitioning regions [1,2]. Collectively, these updated insights depict a disease landscape still defined by substantial clinical and public health challenges, with rising case numbers in several regions and persistent heterogeneity in access to early detection, standardized staging, and evidence-based management [1]. Its high treatment costs further add to this heavy burden, significantly impacting healthcare systems worldwide [3].

In recent years, the landscape of BCa has evolved considerably as advances in molecular pathology, imaging, and therapeutic strategies have refined our understanding of disease behavior. The introduction of updated World Health Organization (WHO) classifications and the recognition of reproducible molecular subtypes have highlighted the biological diversity of urothelial tumors, while simultaneously underscoring the limitations of relying only on conventional histopathology for risk stratification. At the diagnostic level, multiparametric Magnetic Resonance Imaging (MRI) and the novel Vesical Imaging-Reporting and Data System (VI-RADS) have enhanced the accuracy of local staging by improving differentiation between non-muscle-invasive (NMIBC) and muscle-invasive (MIBC) bladder cancer, addressing a long-standing gap in pre-treatment evaluation [2]. NMIBC, which accounts for roughly 75% of incident cases, continues to require intensive and prolonged surveillance because recurrence remains common even after optimal transurethral resection and intravesical therapy. Contemporary evaluation guidelines also underscore persistent disparities in presentation and outcome, noting that women typically experience delayed diagnosis and present with more advanced disease, resulting in worse survival relative to men [2,4,5]. However, despite advances in molecular diagnostics and therapeutics, BCa remains difficult to manage due to its heterogeneous biological behavior and high recurrence rates (Table 1).

Conventional gold standard diagnostic techniques like cystoscopy have inherent invasiveness and sensitivity limits, especially for flat lesions [6]. The invasive aspect of cystoscopy with transurethral resection of bladder tumor (TURBT) can cause patient discomfort and consequences such as bleeding and perforation, yet it is still essential for initial diagnosis and staging [7]. Furthermore, it may not be as sensitive in identifying small, flat lesions and carcinoma *in situ* (CIS), which could result in missed diagnoses and delayed therapy [6]. In addition, urine cytology, a commonly used complementary diagnostic method, has low sensitivity, especially for low-grade cancers [8]. Although numerous urinary biomarkers have been explored, none have yet demonstrated sufficient robustness to replace cystoscopy in routine clinical practice, reinforcing the importance of continued innovation in diagnostic strategies [2].

**Table 1:** Current challenges in bladder cancer diagnosis and therapy.

Challenge	Description	References
High recurrence rate	NMIBC accounts for ~75% of cases	[2,4]
Diagnostic limitations	Cystoscopy (invasive, limited sensitivity for flat lesions), urine cytology (low sensitivity)	[6,7,8]
Treatment toxicity	BCG immunotherapy (effective but systemic adverse effects), chemotherapy (limited by toxicity and resistance)	[9,10]
Delivery barriers	Rapid urinary clearance and the GAG layer reduce intravesical drug bioavailability	[11]

Abb: NMIBC, non-muscle-invasive bladder cancer; BCG, Bacillus Calmette-Guérin; GAG, glycosaminoglycan.

Management strategies vary markedly according to disease stage. Most patients present with NMIBC, which is treated with complete endoscopic resection followed by intravesical therapy, whereas progression to MIBC requires more aggressive interventions such as radical cystectomy or bladder-preserving chemo- and radiotherapy. In advanced or metastatic urothelial carcinoma, treatment options have expanded beyond platinum-based chemotherapy to include immune checkpoint inhibitors and targeted agents, although long-term responses remain limited [2]. Despite advancements in systemic therapies, current evidence indicates that surgery, particularly radical cystectomy, remains the cornerstone of curative treatment for MIBC. Real-world outcomes consistently show that multimodal approaches incorporating surgery with perioperative cisplatin-based chemotherapy, and in selected cases immunotherapy, provide meaningful reductions in progression and improvements in survival [12,13].

Anyway, the effectiveness of established treatments, including immunotherapy, targeted therapy, and chemotherapy, is constrained by systemic toxicity, limited specificity, and the emergence of drug resistance [14]. For example, although Bacillus Calmette-Guérin (BCG) therapy is effective for NMIBC, systemic adverse effects can be problematic, and up to 40% of patients experience recurrence or progression [9]. Similarly, platinum-based chemotherapy continues to form the basis of treatment for MIBC and metastatic disease, yet its usefulness is frequently undermined by toxicity and multidrug resistance [10]. Even the most promising targeted agents and modern immunotherapies face challenges related to patient selection, durability of response, and the development of resistance mechanisms [15]. Taken together, these limitations underscore the need for therapeutic strategies capable of achieving greater precision and sustained efficacy in the management of BCa.

Increasing evidence indicates that optimal outcomes in MIBC are achieved when radical cystectomy is embedded within a multimodal therapeutic framework that includes neoadjuvant cisplatin-based chemotherapy, which improves overall survival, and adjuvant systemic therapies tailored to pathological response and molecular characteristics [16]. Recent translational research also highlights that the molecular heterogeneity of BCa, driven by alterations in FGFR3, TP53/RB1, and PI3K/AKT/mTOR signaling, strongly supports the integration of targeted agents and immunotherapies alongside surgery. In particular, FGFR-altered tumors may benefit from perioperative FGFR inhibitors, while tumors enriched in immune infiltration or high tumor mutation burden (TMB) exhibit enhanced sensitivity to immune checkpoint inhibitors, reinforcing the rationale for neoadjuvant or adjuvant immunotherapy within a surgical pathway [16]. Again, immunotherapy is increasingly incorporated into multimodal strategies since neoadjuvant PD-1/PD-L1 blockade has produced durable pathological complete responses, while adjuvant immunotherapy improves disease-free survival in high-risk patients following cystectomy. These advancements, combined with ongoing efforts to integrate antibody-drug conjugates (ADCs) or gene-therapy-based intravesical approaches for BCG-unresponsive disease, reflect a rapid expansion of multimodal treatment strategies that enhance and complement the curative potential of surgery [16].

Resistance to systemic therapies continues to represent a major barrier to durable disease control. Mechanistic analyses from recent studies demonstrate that non-coding RNAs (ncRNAs), including long non-coding RNAs (lncRNAs) and microRNAs (miRNAs), can deeply influence chemoresistance and recurrence by modulating apoptosis, autophagy, ferroptosis, and DNA damage/repair pathways. Key examples include the LINC00941/IMP2 axis (which drives cisplatin resistance through m6A-dependent stabilization of IPO4 and SLC7A11) [13], the lincRNA-p21/STAT3 regulatory loop (affecting metastatic signaling) [17] and miRNA-driven pathways such as miR-27a/SLC7A11 and miR-101/VEGF-C (modulating cellular sensitivity to platinum-based agents) [12]. Incorporating these current molecular insights together with established surgical and multimodal protocols will be crucial for developing more precise therapeutic algorithms and overcoming the persistent challenge of treatment failure in MIBC.

**Figure 1:**
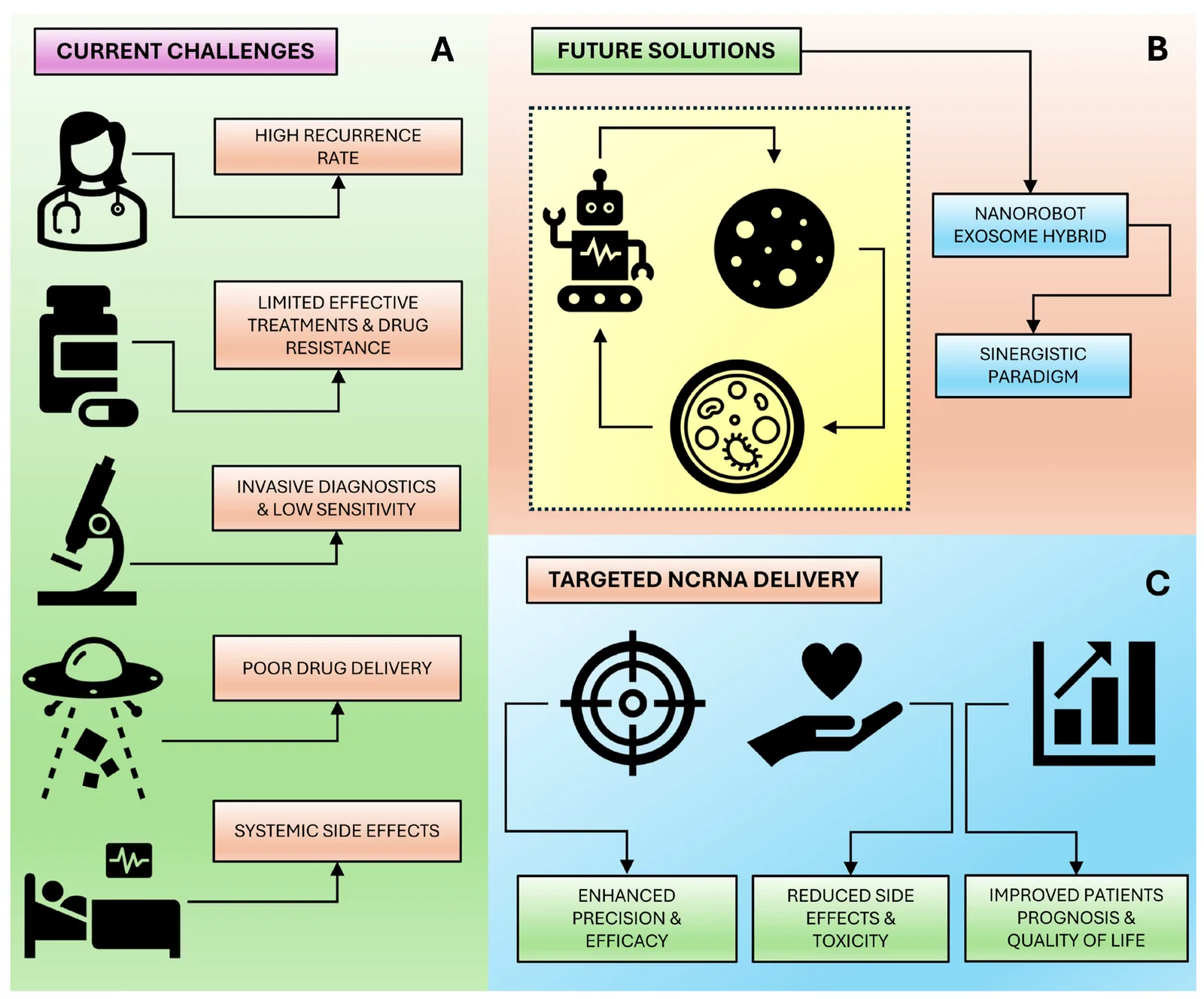
**Integrated nanomedicine for bladder cancer.** (**A**) Current challenges in bladder cancer (BCa) treatment (high recurrence rates, limited therapeutic options, invasive diagnostics, poor drug delivery and systemic side effects) underscore the need for innovative solutions; (**B**) An effective synergistic approach combines nanorobots, exosomes and non-coding RNA (ncRNA) within an integrated nanomedicine framework; (**C**) The goal is to achieve enhanced precision and therapeutic efficacy, minimize toxicity and adverse effects, and finally improve patient prognosis and quality of life. The figure is original and was generated using Microsoft PowerPoint (Version 16.107), utilizing the native vector-based iconography provided within the software. NCRNA, non-coding RNA.

Furthermore, an additional hurdle limiting the efficacy of local treatments remains the delivery barrier. Current intravesical administration methods rely heavily on passive diffusion, which is severely restricted by the rapid washout due to urinary flow and the physical impediment of the glycosaminoglycan (GAG) mucosal barrier. This results in insufficient drug concentration reaching the tumor cells, leading to low bioavailability and high recurrence rates (Fig. 1A).

### 1.2 Nanotechnological Approach in Bladder Cancer

The above-discussed themes point to a field in rapid transition, but still tethered by persistent clinical gaps, most notably in diagnostic precision and the stratification of patients for novel therapies. Overcoming the anatomical hurdles of the bladder and its complex microenvironment remains a primary challenge, providing a compelling rationale for the shift toward bioengineered vesicles and nanotechnology-driven systems designed to transform the efficacy of intravesical delivery. In this regard, an emerging direction in BCa treatment is offered by the development of nanotechnology and molecular therapies. Developed at the nanoscale, nanorobots have emerged as promising instruments for *in situ* diagnosis and targeted medicine delivery, providing the possibility of localized interventions [18]. In order to minimize harm to healthy tissues and lessen systemic side effects, these advanced nanodevices can be designed to precisely identify cancer cells, deliver therapeutic agents to the tumor site, and even carry out minimally invasive surgical procedures [19]. In this perspective, nanorobots, particularly those powered by mechanisms involving urease or external magnetic fields, offer active propulsion and high-precision targeting. This kinetic capability allows them to successfully navigate the bladder environment, overcoming the restrictive GAG layer and counteracting urinary clearance [11]. Given their ability to transport a variety of payloads and navigate precisely throughout the body, they represent adaptable platforms for next-generation BCa treatment [11] (Fig. 1B).

Concurrently, the emerging areas of ncRNA and exosome biology are opening the door for the creation of customized treatments based on the distinct molecular characteristics of individual tumors and offering previously insights into the complex molecular processes that control tumor progression. NcRNAs, such as lncRNAs and miRNAs, are essential for controlling gene expression. They are often dysregulated in BCa, affecting the development, spread, and metastasis of the tumor [20]. Exosomes are tiny extracellular vesicles (EVs) containing a variety of biomolecules, such as proteins, lipids, and ncRNAs, and they have been found to be important intercellular communication mediators in the tumor microenvironment [21]. This makes them potential therapeutic ncRNA delivery vehicles, enabling highly targeted and customized therapies, in addition to being promising biomarkers for early identification and illness monitoring [22].

For instance, the precise delivery of therapeutic ncRNAs to tumor cells could be achieved by combining nanorobotic transport with ncRNA-based therapeutics, thereby overcoming major delivery barriers and enhancing therapeutic efficacy [23,24]. Moreover, nanorobots engineered to detect specific ncRNA signatures could greatly improve diagnostic sensitivity, enabling earlier tumor identification and more accurate disease staging through highly specific liquid biopsy (LB) approaches [18]. Integrating these advanced technologies enables the development of therapeutic strategies capable of addressing the limitations of current treatments and ultimately improving clinical outcomes, prognosis, and quality of life for patients with BCa. By reducing systemic toxicity, enhancing tumor suppression, and mitigating resistance mechanisms that undermine conventional therapies, advances in nanomedicine hold considerable promise for the future of BCa diagnosis and treatment [25] (Fig. 1C).

The central challenge remains ensuring that therapeutic agents reach the tumor in effective concentrations despite urinary clearance and the protective GAG layer of the bladder mucosa. To address these obstacles, this review brings together three distinct yet complementary fields whose integration is likely to shape the future of BCa treatment. Here, we examine the role and potential of nanorobots as active delivery tools, exosomes as natural carriers, and ncRNAs as precise molecular payloads, an analysis that leads to the conceptual foundation for a nanorobot-exosome system designed to enable targeted ncRNA administration. Within this framework, the outcome is not a set of isolated technological paths but a unified and convergent therapeutic strategy. The active propulsion of nanorobots, the intrinsic biocompatibility of exosomes, and the molecular specificity of ncRNAs collectively form an integrated platform capable of overcoming the anatomical and physiological barriers that limit conventional intravesical therapies [18,26]. This synergistic perspective provides the conceptual core for the innovations discussed throughout this review.

## 2 Nanorobots: Overcoming Biological Barriers for Active Drug Delivery

Medical micro- and nano-robots (MNRs) have recently become a very promising technological solution for the detection and treatment of malignant tumors [18]. Precision medicine has advanced significantly thanks to these innovative technologies. By carrying out precise and targeted actions at the cellular level, these advanced devices can overcome a number of inherent limitations associated with traditional cancer diagnostic and therapeutic approaches, including the difficulties in obtaining an early diagnosis that is sensitive enough, the non-specific delivery of drugs, and the emergence of chemoresistance [27]. Despite their value, conventional diagnostic methods frequently have trouble in identifying small lesions early on, which delays management and results in worse prognoses. Additionally, off-target accumulation and the emergence of multidrug resistance sometimes lead to severe adverse effects from systemic drug delivery in conventional chemotherapy, which reduces overall treatment efficacy [10]. Medical MNRs can perform tasks that greatly improve tumor diagnosis sensitivity and specificity, precisely target cancerous tissues, and even function at the level of individual cells. These capabilities stem from their intrinsic properties, including their small size, distant control potential, and ability to carry a therapeutic payload [18]. This suggests a future where interventions can be tailored with growing precision to the specific characteristics and location of bladder tumors.

### 2.1 An Overview of Nanorobot Designs

Various nanorobot kinds, each with distinct properties and modes of action, are being investigated in BCa research at this time and this variety highlights how dynamic and ever-evolving this subject is. The propulsion mechanism is a crucial component supporting autonomous nanorobot systems functioning and clinical application. Nanorobots can effectively transport therapeutic payloads and reach tumor locations by navigating complicated biological settings, such as body fluids and tissues, thanks to the development of a variety of propulsion techniques [18]. Furthermore, metal-organic framework (MOF)-based autonomous nanorobots are being created for active and targeted drug delivery to particular subcellular organelles, such as mitochondria, providing a new degree of precision in cancer treatment [28]. Depending on their intended function, a number of propulsion systems have been developed and others are in the process of being engineered. However, robust clinical translation, long-term biocompatibility and propulsion efficiency optimization remain difficult despite progress. The development of more advanced propulsion strategies that allow real-time tracking, improve control in complex *in vivo* environments and integrate smoothly with therapeutic functions represents a key direction for future research. These innovations are expected to lead the way for increasingly sophisticated and personalized cancer treatments [18] (Table 2).

**Table 2:** Nanorobot propulsion mechanisms and key features.

Propulsion Type	Mechanism	Advantages	Limitations	References
Chemical fuel-based	Catalytic breakdown of urea (z-powered)	High power density, suited for bladder environment	Fuel depletion, byproduct toxicity	[29,30]
Magnetic-driven	External magnetic fields guide nanorobots	Precise, non-invasive control	Requires complex field generation	[31]
Light-driven	Photothermal or photochemical reactions	High spatial accuracy	Limited tissue penetration	[18]
Ultrasound-driven	Acoustic streaming or cavitation	Good tissue penetration	Requires precise control	[18]
Biological propulsion	Uses living cells or natural gradients	High biocompatibility	Difficult scalability	[18,32]
Hybrid systems	Combines two propulsion methods	Enhanced versatility	Complex design	[18]
Autonomous DNA	DNA origami	Versatility, precision	DNA fragility	[33]

- *Chemical fuel-based propulsion.* This group mostly depends on the catalytic breakdown of chemical fuels in the biological environment or enzymatic processes. Despite the high propulsive potential, fuel depletion, possible by-product toxicity and the requirement for certain biochemical conditions are current obstacles [18]. Urease-powered nanorobots have shown strong potential for improving intravesical drug delivery by exploiting the enzymatic hydrolysis of urea to generate gas bubbles that propel them through the urinary environment. This reaction provides the active movement needed to navigate the bladder lumen, making these nanorobots particularly well suited for BCa applications, as demonstrated in preclinical models [29]. Building on this principle, mesoporous silica nanorobots equipped with urease have emerged as a promising platform for bladder-directed therapies. Their porous, sponge-like architecture allows efficient loading of therapeutic agents, while the urease coating provides the enzymatic trigger that exploits urine urea to generate the propulsion required [29].

Urease-driven systems offer quantitative evidence that active propulsion significantly enhances the effectiveness of intravesical delivery. In orthotopic bladder cancer models, nanorobots powered by urease demonstrated an increase in tumor deposition (approximately an eightfold improvement, as measured by *in vivo* Positron Emission Tomography-PET imaging) compared to their non-motile counterparts [29]. Beyond simple accumulation, these platforms showed a clear ability to penetrate the tumor mass, with measurable distribution reaching depths of about 100 μm from the luminal surface [29]. As a result, the tumor size was reduced by 90% when these nanorobots were utilized for radionuclide treatment carrying a radioactive isotope as the payload [29]. Supporting again these findings, urine-derived exosome vesicles powered by urease exhibited a motility advantage over those reliant on Brownian motion. Thanks to self-sustained propulsion, these vesicles bypassed the dense GAG barrier and resisted micturition-induced washout, achieving deep mucosal and intratumoral infiltration. The mean velocity of these vesicles went from 1.5 ± 0.1 to 5.6 ± 0.3 μm/s, which facilitated a significant increase in translocation through bladder mucus, rising from 17.6 ± 1.7% to 55.1 ± 4.2% (about a 3.1-fold improvement). This enhanced mobility facilitated detectable signal diffusion to a depth of approximately 120 μm within the 3D bladder tumor spheroids [30]. Furthermore, *in vivo* data confirmed that this self-propulsion mechanism extended bladder retention beyond four hour, whereas free payloads were rapidly cleared within the first hour of administration [30]. This enhanced penetration translated into larger tumor accumulation and sonodynamic efficacy, ultimately yielding better survival outcomes [30]. This demonstrates that urease engineered nanorobots represent a viable and effective platform for delivering therapeutic agents in BCa.

- *External field-driven propulsion.* This method avoids the requirement for on-board fuel by using external energy sources to regulate MNR movement. Important techniques include light, ultrasound and magnetism. (a) Light-driven propulsion uses light energy to trigger photothermal or photochemical processes that result in asymmetric heating or bubble formation. High spatial accuracy and remote control are provided, although the depth of light penetration in tissues limits this; (b) Ultrasound-driven MNRs are propelled by using ultrasonic vibrations to create cavitation bubbles or acoustic streaming. Although this technique has good tissue penetration and biocompatibility, it necessitates precise ultrasonic parameter control; (c) Magnetic-driven devices are generated by incorporating magnetic elements into MNRs. Consequently, external magnetic fields may be used to precisely and remotely control them. Although complex field creation may be needed for elaborate operations, this offers great tissue penetration and non-invasiveness, making it a very attractive path for *in vivo* applications [18,31].
- *Biological propulsion.* Specific MNRs rely on biological systems or mechanisms for movement. This includes designs that exploit natural biochemical gradients or physiological flows, or approaches in which therapeutic agents are coupled with living cells such as bacteria or sperm. While these strategies offer intuitive navigation and excellent biocompatibility, they also present challenges in terms of control and scalability. Bacteria-assisted delivery platforms provide a particularly interesting rationale for actively guided intravesical therapies. Wang and colleagues demonstrated that engineered commensal *Lactobacillus* strains can achieve stable urothelial adhesion and deep penetration into bladder tissue without eliciting inflammatory responses. By harnessing their intrinsic tropism and biomechanical compatibility, these microbial vectors can be tailored for persistent retention within the bladder environment. Such findings highlight the potential of biologically driven propulsion systems to overcome urothelial barriers and enhance spatial retention and payload delivery in restrictive intravesical settings [32].
- *Hybrid propulsion systems.* These systems integrate two or more processes to get over the drawbacks of single propulsion techniques. A magnetic-chemical hybrid system, for instance, could improve performance and versatility in dynamic biological environments by providing both continuous propulsion and accurate steering. In order to provide more control and flexibility, these systems seek to maximize advantages of each mechanism while minimizing its disadvantages [18].
- *Autonomous DNA nanorobots.* DNA nanorobots are an exciting development in medicine because they can be programmed to act as smart and autonomous delivery systems. Using DNA origami (the ability to fold DNA into specific shapes) nanostructures that travel through the body, sense their surroundings and release medication only when they reach a target site, can be engineered. These nanorobots use locks that open in response to specific triggers, like the presence of certain miRNAs or changes in pH, which helps ensure the drug hits the tumor without affecting healthy tissue [33]. These systems are versatile enough to act as both diagnostic sensors and drug carriers, changing their shape to perform tasks once they find targets. However, translating this technology from the lab to the clinic is challenging since DNA is naturally fragile and can be broken down by enzymes or triggered by the immune system. Researchers are now testing ways to protect them, such as wrapping the nanorobots in a lipid layer or using chemical coatings and these steps are crucial to ensure that these nanorobots remain stable and effective when operating in difficult environments, such as the bladder [33].

### 2.2 Propulsion Performance Benchmarks

Performance benchmarks of various nanorobot platforms are influenced by device dimensions, medium viscosity, ionic strength and actuation method. As a consequence, context-dependent comparisons are essential when evaluating bladder-specific outcomes against broader standards [19]. In terms of propulsion, external-field systems typically provide superior controllability compared to purely chemical alternatives [18,19,31], however, showing specific advantages as well as limitations. Light-driven systems often operate within the ~1–10 μm/s range, though their utility is restricted by poor optical penetration through biological tissues [19]. Ultrasound-driven systems, frequently reported at speeds of ~5–10 μm/s, offer the advantage of deep tissue access (reaching several centimeters), yet precise navigation in complex environments remains a limiting factor [18,19,31]. Finally, magnetic approach offers a robust balance of wireless maneuverability and high tissue penetration. Depending on the specific geometry and magnetic field programming, speeds can range from ~1 to 300 μm/s [19,31]. The performance of chemically-powered systems, conversely, is often expressed via effective diffusion coefficients (D_t_) rather than linear velocity and within this category, urea- and glucose-fueled nanorobots operate in the ~0.5–2 mm^2^/s range, while fuels such as H_2_O_2_, glutathione (GSH) and lactate can drive speeds of ~1–6 mm^2^/s, depending on the local substrate availability [19].

These performance benchmarks, therefore, explain how active and motile carriers can extend targeting capabilities beyond limits of ligand-receptor recognition and, furthermore, offer a strategy to overcome the low tumor delivery efficiency observed with conventional passive nanocarriers [19,34].

### 2.3 Nanorobots as a Vector for ncRNAs and Nanoparticles

Nanorobots are being researched as efficient vectors for the targeted delivery of therapeutics to BCa, in addition to their use in diagnosis. Numerous payloads, such as drugs, nucleic acids, and sensing molecules, can be delivered straight to the tumor location via these tiny devices [35]. Nanorobots and nanoparticles can greatly improve BCa therapy outcomes by strengthening treatment delivery efficacy and enabling precise therapeutic performance [27]. Notably, the nanorobot functions as an active propulsion system, making it an ideal carrier for complex molecular cargos, including ncRNAs and natural nanoparticles, like exosomes. In particular, when given intravesically, self-propelled nanorobots have demonstrated improved diffusion and mixing capabilities inside the urine, providing an advantage over passive nanoparticles or traditional medications [29]. In order to reach all tumor cells, particularly those in hard-to-reach places, this active propulsion enables more even dispersion across the bladder lumen and improved penetration into the bladder wall [29].

With the employment of autonomous movement and navigation of MNRs within biological media, this motile-targeting technique enables the highly precise delivery of therapeutic agents to particular tumor locations, potentially lowering the necessary drug dosage and minimizing systemic side effects [34]. Additionally, the creation of autonomous nanorobots that can actively deliver drugs to the mitochondria is a promising approach that has demonstrated enhanced anticancer effects and metastasis suppression in preclinical research [28]. Targeting mitochondria, which are essential for apoptosis and cellular metabolism, is a potential way to overcome medication resistance and cause more effective cancer cell death [36]. This capacity to carry and actively deliver diverse payloads, including gene therapy components (ncRNA) and exosomal vehicles, is central to the proposed hybrid strategy for BCa.

The potential for active propulsion in the bladder is further validated by dual-source catalytic systems that harness urea and hydrogen peroxide to drive autonomous motion. Dual-fuel architectures suggest that stronger propulsion directly correlates with more efficient barrier penetration and deeper tumor infiltration. For instance, sea urchin-like Au-Pt@urease nanomotors exhibited a significant increase in mobility when exposed to a combination of urea and H_2_O_2_, reaching a D_t_ of 1.67 ± 0.11 μm^2^/s at 500 mM urea plus 3.2 mM H_2_O_2_. This enhanced kinetic energy enabled the motors to navigate an artificial mucus barrier with high efficiency, achieving 47.4% and 87.5% translocation at one and five hours, respectively [37]. Orthotopic model data confirmed that these dual-fuel conditions resulted in a broader intratumoral distribution compared to controls where only a single or no fuel was utilized [37]. Progress in enzyme-powered nanomotors provides an additional mechanistic framework for the hybrid strategy. Chen and colleagues demonstrated that the dual-loaded UG-M@Gem construct achieves directed motion within physiological urinary environments, shifting from Brownian diffusion to active propulsion, then surpassing passive carriers. These structurations are designed to protect the enzymes, allowing for sustained catalytic activity even under harsh conditions. By maintaining this functionality, the platforms can achieve continuous urea-driven propulsion, which ultimately facilitates deep penetration into the bladder wall following intravesical instillation [37,38].

These findings provide a strong rationale for an hybrid model, showing that incorporating an active propulsion module, such as a nanorobot, extends the spatial reach and functional engagement of exosomal vectors, thereby optimizing ncRNA delivery within restrictive bladder microenvironments. At the same time, they reinforce the broader principle that active propulsion can overcome the diffusion-limited barriers, particularly tissue penetration and drug retention, that the nanorobot-exosome platform is specifically designed to address.

### 2.4 The Use of Nanotechnology for Insights in Bladder Cancer Diagnosis

The use of nanotechnology, including nanorobots and nanoparticles, holds potential for improving BCa diagnostics by enhancing *in vivo* imaging and thereby facilitating more accurate detection and assessment of malignant lesions [27]. Fluorescent or photodynamic diagnostic methods based on nanotechnology are being investigated to increase the detection sensitivity of NMIBC in comparison to conventional white light microscopy [39]. According to Daneshmand and colleagues [40], hexaminolevulinate (HAL) fluorescence cystoscopy has demonstrated higher detection rates for NMIBC, especially for flat lesions (like CIS) that are frequently overlooked by conventional white light cystoscopy. In order to guide more accurate tumor removal, cancer-targeting nanoparticles that are made to convey imaging agents can greatly help identify tumors during cystoscopy. Additionally, the establishment of nanoparticle-based techniques to extract cancerous cells or their subcellular constituents from urine samples holds promise for early recurrence identification and follow-up surveillance [39]. By using exosomal or circulating tumor DNA (ctDNA) from urine, this LB technique provides a highly sensitive and non-invasive way to track the progression and recurrence of the disease, potentially eliminating the need for repeated invasive cystoscopies [41]. In preclinical animal models, multimodal mesoporous silica nanoparticles with both fluorescence and MRI contrast properties have shown promise in improving BCa staging and real-time monitoring [42]. For individualized treatment planning, such multimodal imaging capabilities could enable thorough tumor characterization, providing accurate details on tumor size, location, and metabolic activity [43].

### 2.5 Future Challenges and New Directions

Although preclinical research has shown promising results, there are still a number of major obstacles to overcome before BCa may be treated with nanorobot technology [27]. For MNRs to function effectively, factors pertaining to the physiological environment of the bladder and driving mechanisms must be taken into account. Consistent and reliable nanorobot performance is severely hampered by the dynamic nature of the bladder, which includes urine flow, pH fluctuations, and the presence of inhibitory compounds. This necessitates active propulsion mechanisms, such as urease-powered or magnetic systems, to overcome the inherent challenges. Additionally, to minimize any adverse effects on healthy tissues and physiological functions, the materials used to construct nanorobots must exhibit low toxicity, strong biocompatibility, degradability, and the capacity to be safely cleared from the body [18]. Additionally, one major obstacle is still getting beyond biological barriers in the bladder, like the mucus layer and the vascular barrier, as well as the GAG layer on the bladder wall, which prevents medications from penetrating [18]. Novel surface alterations and nanorobot propulsion systems are needed to get past these obstacles, but research on the translational potential of nanorobots to treat BCa is still in its infancy [29]. To understand why these active systems are essential, it is necessary to first examine the limitations of current clinical standards, evaluate passive carriers and detail how traditional drug delivery is constrained by both temporal and physical barriers.

#### 2.5.1 Intravesical Barriers and the Limits of Passive Carriers

The continuous production of urine and periodic voiding cycles result in rapid drug dilution and washout, significantly reducing the dwell time of therapeutic agents [11,44]. As a consequence, passive diffusion often fails to provide the sustained exposure necessary to effectively treat lesions. The primary physical obstacle of bladder is a dual-layered permeability barrier that in fact blocks passive transport. Firstly, the GAG/mucus layer acts as a selective filter, trapping agents through both size exclusion and surface interactions, which often prevents even nanoparticles from reaching the underlying tissue [11,44]. Beneath this, the urothelium provides a second barrier line thanks to specialized cells that are tightly sealed by intercellular junctions, creating an impermeable barrier to most therapeutic agents [11,45]. Together, these two layers form a robust structural defense that limits the efficacy of passive delivery methods and causes a drop in drug concentration across the urothelium, with levels in underlying tissue that can be even an order of magnitude lower than in the bladder lumen [11,45]. Hence, passive diffusion typically results in only superficial exposure, leaving deeper tumor layers inadequately treated. Even with high luminal concentrations, the combined effects of urine washout and poor tissue permeability compromise therapeutic outcomes [11,44]. Therefore, the failure of passive carriers necessitates penetration-enhanced delivery methods to reach deeper tumor cells.

In general, the intravesical environment creates a dual-barrier to drug delivery consisting of (a) time constraints (due to continuous urine washout) and (b) physical barriers (the GAG/mucus layer and the urothelium). These factors demonstrate why passive nanocarriers are inadequate, then necessitating the development of systems engineered for either prolonged retention and active barrier penetration to achieve therapeutic efficacy [11,44,45].

#### 2.5.2 The Active Advantage of Nanorobots in Overcoming Barriers

A key distinction between actively propelled MNRs and conventional passive nanoparticles lies in their ability to overcome the above-mentioned dual-barrier consisting of rapid urinary clearance and the GAG/urothelium layer. While passive carriers rely solely on diffusion (making them susceptible to dilution and voiding), MNRs can sustain directed motion to maintain proximity to the bladder wall and actively navigate the GAG coating [18,29]. This propulsion-driven behavior increases the probability of drug deposition at the tumor surface and enables a level of tissue engagement that passive nanoparticles cannot achieve [29]. Specifically, intravesical platforms powered by urease have demonstrated extended residence times, remaining detectable for approximately four hours, while passive therapeutic payloads are typically cleared from the bladder within the first hour of administration [29,30].

However, considering the GAG/urothelium layer as the sole obstacle would be reductive. The therapeutic advantage of MNR-based propulsion lies also in its ability to navigate toward irregular tumor surfaces, penetrate deeper tissue planes and access poorly perfused niches that passive particles fail to reach [18,29]. Integrating this perspective underscores that tissue depth, architectural complexity and heterogeneous lesion morphology are critical determinants of therapeutic accessibility, factors for which active propulsion may provide a significant advantage.

#### 2.5.3 Systemic Challenges and Clinical Translation

Despite these advantages, ensuring the effectiveness of nanorobots within the intricate human body presents further obstacles [27]. The effectiveness and bioavailability of nanorobots can be considerably diminished by reticulo-endothelial system clearance, opsonization by immune proteins, and enzyme degradation [46]. Before clinical application can be achieved, it is imperative to have a better understanding of the precise mechanisms of action of nanotechnology-based approaches as well as the metabolism, biodistribution, and clearance of nanoparticles (and consequently, nanorobots) [27]. Thorough *in vivo* research in animal models is essential for determining the best dose schedules and evaluating long-term safety and efficacy. In summary, the abilities of nanorobots for active targeting and high tissue penetration make them ideal candidates for the delivery of complex molecular payloads. Specifically, nanorobots represent the means to overcome the challenges posed by the bladder microenvironment, ensuring the optimal accumulation of therapeutic agents (such as those loaded in exosomes or ncRNA-based) directly into tumor cells, a crucial advantage over passive diffusion or conventional administration.

## 3 Exosomes: Precision Biomarkers and Natural Carriers for ncRNA

Exosomes are secreted EVs with a key role as mediators of intercellular communication across physiological and pathological contexts [47]. They are nanoscale EVs of 30–150 nm in diameter, generated through the inward budding of the endosomal membrane and subsequently released when multivesicular bodies fuse with the plasma membrane. They are secreted by virtually all cell types, and their presence is evidenced across multiple biological fluids, including blood, urine, saliva, cerebrospinal fluid, and amniotic fluid, reflecting their stability and broad physiological distribution. Because their lipid bilayer protects internal cargo from degradation, exosomes efficiently transport a wide spectrum of bioactive molecules such as proteins, lipids, DNA fragments, messenger RNAs (mRNAs), miRNAs, and lncRNAs, which are selectively sorted into intraluminal vesicles through Endosomal Sorting Complex Required for Transport (ESCRT)-dependent or ESCRT-independent (mediated by tetraspanins like CD63) mechanisms [48,49]. At the functional level, exosomes act as potent mediators of intercellular communication, transferring their molecular cargo to neighboring or distant recipient cells and modulating gene expression, signaling pathways, immune responses, and metabolic programs. This communication role is further underscored by evidence showing that exosomes can influence both innate and adaptive immunity, participate in antigen presentation, and alter the behavior of stromal and immune cells within pathological microenvironments [49,50].

**Figure 2:**
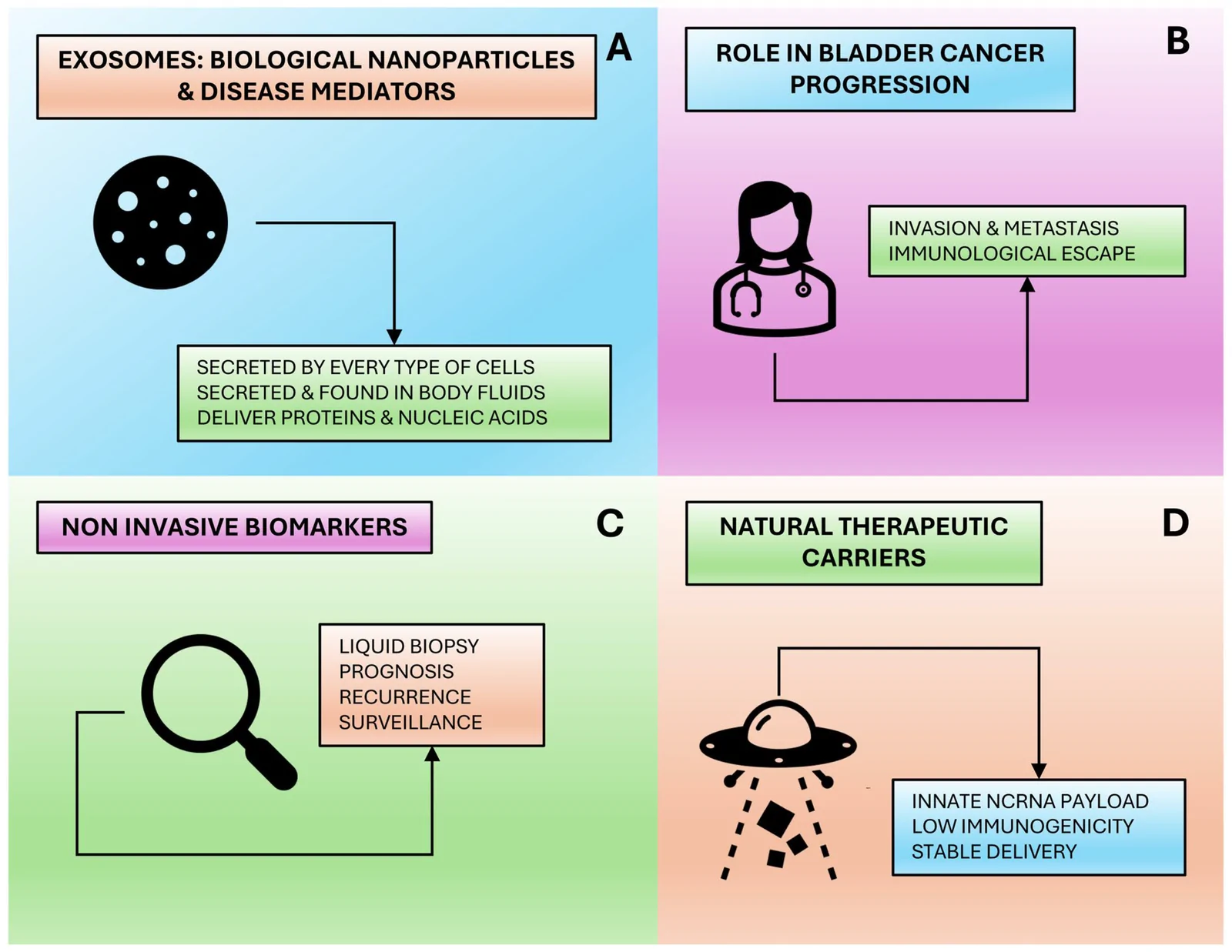
**Exosomes as biological nanoparticles and their role in bladder cancer.** (**A**) Exosomes, secreted by most cell types, function as biological nanoparticles and key mediators of disease; (**B**) They play a pivotal role in bladder cancer (BCa) progression, including invasion, metastasis and immune evasion; (**C**) Exosomes hold potential as non-invasive biomarkers for prognosis and recurrence monitoring; (**D**) Their intrinsic properties make them highly promising as natural carriers for non-coding RNA (ncRNA), offering low immunogenicity and stability in cargo delivery. The figure is original and was generated using Microsoft PowerPoint (Version 16.107), utilizing the native vector-based iconography provided within the software. NCRNA, non-coding RNA.

In BCa, where urine directly interfaces with the tumor microenvironment, exosomes serve as accessible and robust markers of tumor biology (Fig. 2A) [49,50,51]. Their intrinsic stability, combined with the continuous shedding of vesicles into the urine, provides a platform for non-invasive and repeatable molecular profiling. By capturing both primary drivers of disease and clinically actionable biomarkers, exosomal cargo offers a high-fidelity window into the evolving landscape of tumor [48,50,51].

Specifically, BCa-derived exosomes modulate angiogenesis, epithelial-mesenchymal transition (EMT), immune evasion, and chemoresistance through cargo such as miR-184, miR-152-3p, HOTAIR, and LUCAT1, thereby actively promoting tumor progression. Beyond nucleic acids, exosomal proteins including α1-antitrypsin, apolipoprotein E, MMP7, MMP12, and histone H2B1K have shown diagnostic discrimination between BCa and benign or inflammatory conditions, supporting their utility in early detection and risk stratification [51]. Again, in the tumor context, exosomes can help to control immunological escape, which enables tumor cells to avoid being recognized and eliminated by the host immune system [48] (Table 3). It has been demonstrated that exosomal miR-184 (released from BCa cells) promotes cancer growth by causing immune escape and targeting the AKR1C3 gene [52]. Additionally, it has been reported that exosomal lnc-TAF12-2:1 promotes BCa progression by sponging miR-7847-3p, thereby upregulating the ASB12 axis to drive tumorigenesis [53]. Exosomes from BCa that carry the protein EDIL-3/Del1 also aid in the spread of the disease [54]. Furthermore, exosome-derived LINC00960 and LINC02470 increase the aggressiveness of BCa cells and facilitate EMT, a critical step in metastasis [55]. Exosomes can drive macrophages toward an immunosuppressed state that accelerates BCa progression [56] largely through exosomal cargo that converts them into tumor-associated macrophages (TAMs), thereby creating a pro-tumorigenic microenvironment [57]. In BCa, serum exosome-derived circSCL38A1 has also been linked to advanced tumor progression through a mechanism involving the ILF3/TGF-β2 signaling axis [58]. These observations, in general, highlight the intricate and varied role that exosomes play in the initiation and spread of BCa (Fig. 2B).

**Table 3:** Roles and applications of exosomes in bladder cancer.

Function	Examples	References
Tumor progression	miR-184, EDIL-3, lnc-TAF12-2:1, circSCL38A1	[52,53,54,58]
EMT and aggressiveness	LINC00960, LINC02470	[55]
Immunosuppression	Macrophage polarization	[56]
Biomarkers	Urinary exosomal miRNAs	[59]
Therapeutic delivery	LncRNA MEG3	[60]

Abb: EMT, epithelial-mesenchymal transition; miRNA, microRNA; lncRNA, long non-coding RNA.

### 3.1 Exosomes as Natural ncRNA Delivery Vehicles

Exosomes are increasingly recognized as promising delivery vehicles for therapeutic applications in BCa due to their distinctive lipid-bilayer structure and biological properties [21]. Their inherent biocompatibility, low immunogenicity, and ability to stably transport endogenous ncRNAs provide clear advantages over synthetic nanoparticles such as liposomes, enhancing treatment efficacy while potentially reducing adverse effects [26,48]. Compared with other delivery systems, exosomes offer high cargo stability, natural compatibility with biological barriers, and the capacity to carry diverse payloads, including proteins, nucleic acids, and small molecules [26,61]. These features make them particularly suitable for gene-based therapies; for example, exosome-mediated delivery of artificial circRNAs is being explored as a novel approach for BCa [62]. Furthermore, studies in other malignancies have shown that engineered exosomes can serve as effective carriers for lncRNAs, suggesting similar opportunities in BCa [60]. By loading specific therapeutic agents and modifying their surface to target malignant cells with precision, exosomes can increase drug accumulation at tumor sites and limit off-target exposure, supporting their potential role in personalized treatment strategies [63].

A further consideration when employing exosomes as ncRNA carriers is the challenge of endosomal and lysosomal sequestration after cellular uptake. This bottleneck can limit the intracellular bioavailability of the therapeutic payload, but in principle, engineered modifications to the exosomal membrane, such as pH-responsive lipids or fusogenic peptides, could promote endosomal escape and enhance cytosolic delivery [63]. A hybrid system concept introduces the additional opportunity since the nanorobotic component could be designed to trigger localized physical or biochemical cues (photothermal or magnetic stimulation) that facilitate membrane destabilization and release of the ncRNA cargo into the cytoplasm [18]. Such combined strategies may significantly improve the functional effectiveness of exosome-mediated ncRNA therapy within the BCa microenvironment.

### 3.2 Exosomes as Non-Invasive Biomarkers and Therapeutic Targets

As non-invasive diagnostic and prognostic indicators for BCa, exosomes have great potential because of their capacity to mirror the molecular status of their parental cells [21]. Their extensive distribution throughout the body, tissue specificity, high abundance and strong stability in body fluids make them easily accessible for investigation [21]. Exosomes, which can be used as biomarkers for BCa diagnosis, prognosis, and surveillance, have been found to be abundant in urine [27]. Urinary exosomes are excellent candidates for LB in BCa because of the non-invasive nature of urine collection and the relative stability of exosomal cargo [22]. Urinary exosomal miRNAs, for example, have demonstrated potential as biomarkers for BCa detection and for differentiating between MIBC and NMIBC [59]. Significantly, even after full clinical downstaging after therapy, urine exosomes from BCa patients have been shown to retain a residual cancer phenotype, indicating their potential for identifying minimal residual disease and for predicting recurrence [64]. This feature is especially helpful for tailored surveillance plans since it enables prompt intervention and early relapse detection. When examined in blood and urine samples, exosomal ncRNAs have generally shown excellent diagnostic effectiveness in identifying BCa [65] as is the case with the lncRNA BCYRN1, evaluable in serum exosomes [66]. These results prove the potential of exosomes as a useful instrument for BCa treatment using non-invasive LB (Fig. 2C).

Diagnostic applications of exosome-based LB continue to evolve, balancing clinical promise with inherent methodological hurdles. While studies in cancer demonstrate that specific miRNA signatures can reliably stratify disease stages, they also highlight the urgent need for standardized workflows to ensure clinical scalability. To this end, innovative approaches, such as dual-protein recognition and *in situ* miRNA profiling, are addressing signal-to-noise limitations by enhancing vesicle specificity. These advancements align with next-generation analytical frameworks, where the simultaneous detection of orthogonal molecular features (combined protein-miRNA assays) effectively mitigates the confounding effects of vesicle heterogeneity [67]. Furthermore, selective barcoding and fusion-based readouts now allow for the enrichment of tumor-specific vesicles, significantly reducing background interference [68]. As imaging resolution and assay sensitivity improve, the reliability of these biomarkers hinges on the rigorous calibration of pre-analytical variables [69]. Eventually, transitioning from proof-of-concept to robust clinical implementation requires a cohesive strategy that integrates technical sophistication with disciplined experimental design [47,70].

The accurate use of exosomes as biomarkers, however, requires resolving several technical problems in current analytical methods. Despite the potential of tumor-enriched ncRNAs, the field remains constrained by intrinsic vesicle heterogeneity and the challenge of distinguishing tumor-derived signals from the non-malignant background. These limitations are underscored by findings that exosomal cargo is highly dynamic and fluctuating in response to cellular stress and disease progression. Furthermore, isolation protocols (ultracentrifugation, density-gradient fractionation, size exclusion chromatography-SEC) introduce technical noise that significantly impacts yield and molecular composition [70]. High-sensitivity platforms demonstrate that even advanced assays struggle with pre-analytical variability and sample complexity when applied to clinical cohorts [47,68]. More importantly, the presence of non-vesicular, Argonaute-associated miRNAs further complicates data interpretation. Consequently, robust clinical translation demands rigorous standardization of isolation workflows and the implementation of stringent experimental controls to ensure that identified molecular signatures are truly reflective of the disease state.

Exosomes are also being investigated as potential treatment targets for BCa in addition to their function as biomarkers [59], and the discovery of exosomal protein interactors opens up new therapeutic options [71]. To prevent tumor growth and metastasis, methods that target the synthesis or release of tumor-derived exosomes, eliminate them from circulation, or prevent recipient cells from absorbing them, are being researched [72]. For example, utilizing several inhibitors to decrease exosome biogenesis or release may reduce their pro-tumorigenic effects [73,74]. As an alternative, new strategies are investigating the use of exosome sponges to counteract circulating carcinogenic exosomes [48,74]. Because exosomes can affect the tumor microenvironment and mediate signaling between tumor cells, it may be therapeutically advantageous to target exosomes in order to interfere with these processes. Systems-level studies further underscore why exosomes represent compelling therapeutic targets in BCa. Shen and colleagues demonstrated that exosomal gene networks strongly influence tumor behavior, identifying a 15-gene signature that differentiates prognostic groups and highlighting THBS1 as a key exosomal effector whose silencing suppresses metastatic traits. These findings reveal exosomal signaling as a coordinated driver of stromal interaction and immune escape, reinforcing the rationale for approaches that directly disrupt or repurpose exosome-mediated communication, including nanorobot-guided and ncRNA-loaded platforms [75]. Additional evidence from Huang and colleagues shows that gemcitabine-resistant BCa cells remodel their metabolic and epigenetic landscape through the exosomal export of the histone variant H3.2/H3C14. This dual-function EV system disseminates resistance-promoting signals while removing suppressive factors, thereby sustaining chemoresistance. Targeting these exosome-dependent pathways, either by stabilizing inhibitory histone pools or by delivering remedial ncRNAs, offers a clear therapeutic opportunity that hybrid exosome-nanorobot systems are well positioned to exploit [76].

### 3.3 Potential Exosomal Modifications for Nanorobot Integration

The key role of exosomes as mediators in BCa progression and as LB diagnostic tools is now well consolidated [48,49], and their nature as biological nanoparticles, capable of stably carrying endogenous ncRNAs, qualifies them as ideal vectors for nanomedicine [21,26] (Fig. 2D). However, to optimize the administration of modified or enhanced exosomes to specific tumor sites in the bladder, it is necessary to set up an active guidance and targeting mechanism. This is where the need to integrate nanorobots emerges, providing the engine required for targeted exosomal delivery [19,35]. For the proposed hybrid system to function, exosomes must be engineered to interface with the nanorobot, and this integration requires exploring modifications on their surface (functionalization) that facilitate stable and reversible attachment to the nanorobots [60,63]. Potential strategies include conjugating magnetic tags or specific surface markers onto the exosomal membrane, which could serve as a recognition site or attachment point for the nanorobots to actively transport exosomes to the desired tumor location [31]. Additionally, established bioconjugation techniques such as copper-free click chemistry, streptavidin-biotin bridging, or peptide-lipid anchoring could provide stable yet reversible interfaces between the nanorobot chassis and engineered exosomal membranes. These approaches have been widely applied in nanoscale assembly and offer tunable binding affinities compatible with physiological conditions, thereby enabling controlled loading and release of the exosomal cargo [63].

## 4 Non-Coding RNA: The Therapeutic and Diagnostic Target for Bladder Cancer

**Figure 3:**
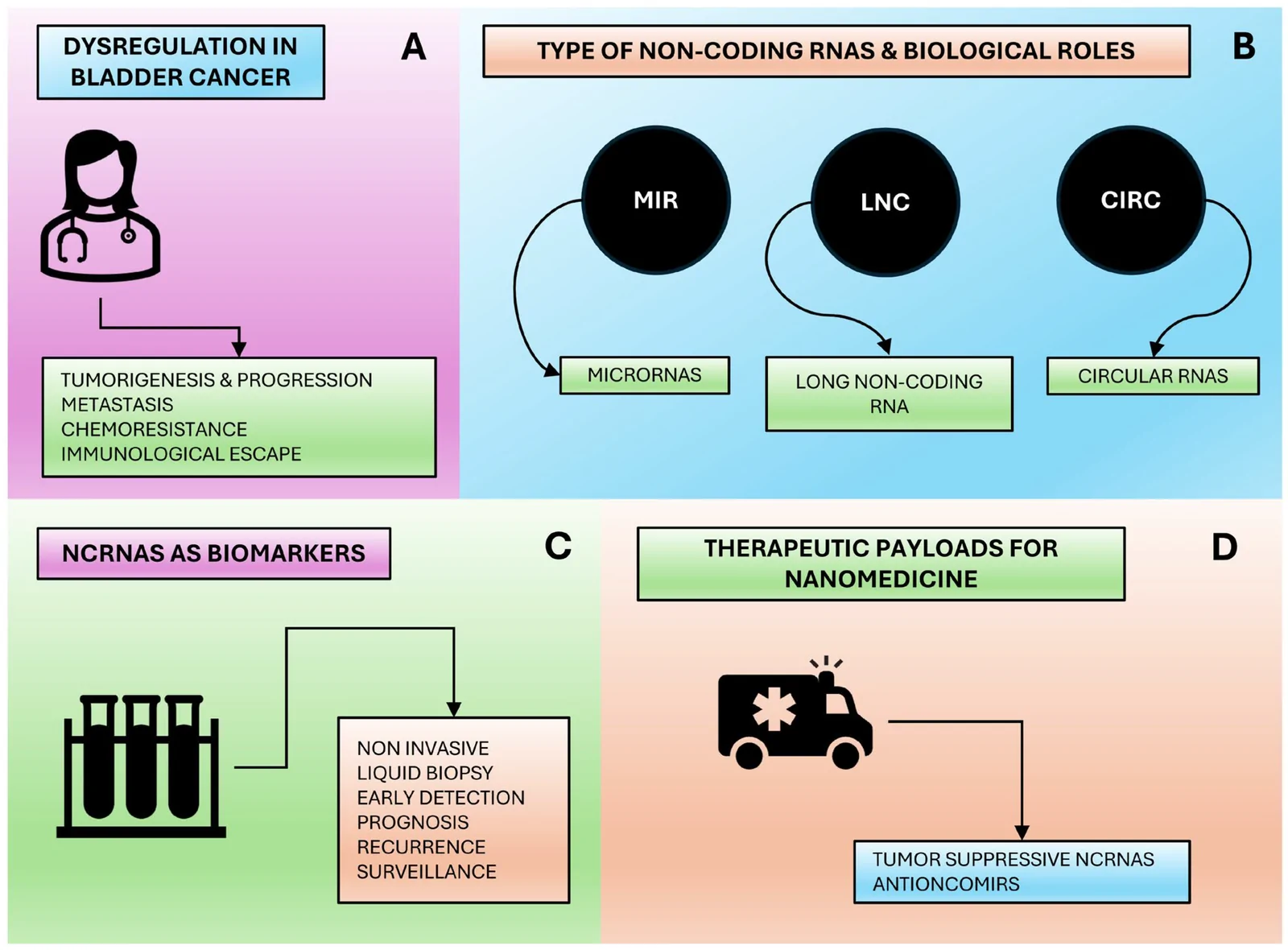
**Types and biological roles of non-coding RNAs in bladder cancer.** (**A**) Non-coding RNAs (ncRNAs), whose dysregulation is strongly associated with bladder cancer (BCa), influence tumorigenesis, cancer progression, and immune evasion; (**B**) Overview of key ncRNA classes: long non-coding RNAs (lncRNAs), microRNAs (miRNAs), and circular RNAs (circRNAs); (**C**) Due to their inherent structural stability and low immunogenicity, ncRNAs are increasingly recognized as promising biomarkers; (**D**) Exploiting their tumor suppressive funtions, ncRNAs are also employed as natural therapeutic agents. The figure is original and was generated using Microsoft PowerPoint (Version 16.107), utilizing the native vector-based iconography provided within the software. NCRNA, non-coding RNA; MIR, microRNA; LNC, long non-coding RNA; CIRC, circular RNA.

NcRNAs, which include miRNAs, lncRNAs, and circRNAs, have emerged as important players in the onset and progression of BCa [77] (Table 4). These non-protein-coding RNA molecules act directly at the RNA level, in contrast to mRNAs, which are translated into proteins [78], and play a role in a variety of biological functions, including cell proliferation, invasion, and drug resistance (Fig. 3A). MiRNAs function by binding to target mRNA regions and triggering translational repression or mRNA degradation, then controlling gene expression post-transcriptionally [79]. In this context, they can act as both tumor suppressor genes and oncogenes based on the genes they target. They are commonly dysregulated in human malignancies, including BCa, and their abundance in body fluids makes them useful biomarkers for early cancer detection.

**Table 4:** Non-coding RNAs in bladder cancer.

ncRNA Type	Role	Examples	References
miRNAs	Tumor suppressors or oncogenes	miR-145 (downregulated), miR-9 (upregulated)	[80,81]
lncRNAs	Regulate proliferation, apoptosis, and drug resistance	UCA1, SNHG1, MIR31HG	[82,83,84]
circRNAs	Act as miRNA sponges, regulate signaling	circRNA MYLK, circRNA ACVR2A	[85,86]

Abb: miRNA, microRNA; lncRNA, long non-coding RNA; circRNA, circular RNA.

LncRNAs are molecules longer than 200 nucleotides, performing a variety of regulatory tasks such as post-transcriptional processing, gene transcription, and chromatin remodeling [87]. They are important regulators of gene expression as well, and their abnormal expression has been linked to the development of many malignancies, including BCa, where they might affect prognosis and contribute to drug resistance [88]. Since their dysregulation can have a substantial effect on carcinogenic pathways, they are desirable targets. Finally, circRNAs, a relatively novel type of ncRNAs, perform important functions in normal physiology and development, and they are frequently dysregulated in BCa, implying their participation in the disease [89]. CircRNAs, in contrast to linear RNAs, have a covalently closed loop structure, which makes them extremely robust and protected from exoribonuclease degradation [90]. Their enhanced stability and their various roles underscore their strong potential as diagnostic and therapeutic targets in BCa (Fig. 3B).

### 4.1 The Deregulation of MicroRNAs in Bladder Cancer

MiRNAs, which are short ncRNA molecules, are critical for influencing gene expression and play an important role in the genesis and progression of BCa [91]. Dysregulation of miRNAs in BCa is linked to a variety of cellular processes, including cell cycle arrest, apoptosis, proliferation, metastasis, and drug resistance [92]. As a result, miRNAs have the potential to improve BCa diagnosis, prognosis, disease monitoring, and tailored treatment [93] (Fig. 3B,C). Their long-term stability in body fluids such as urine and blood makes them excellent potential non-invasive markers for early detection, monitoring treatment progress, and predicting outcomes in patients with BCa [91]. Because BCa has a high recurrence rate, the ability to identify particular miRNA signatures in readily available biofluids provides a less invasive option to standard biopsy, and this could be especially advantageous for surveillance [94]. Furthermore, miRNAs have the potential to improve immunotherapy efficacy and facilitate the formulation of tailored treatment plans. It is worth noting that urine miRNAs can also be used to diagnose BCa, with particular miRNA patterns demonstrating excellent sensitivity and specificity [91]. For example, miR-9 overexpression has been linked to a more aggressive BCa phenotype and an increased risk of disease progression [81]. On the other hand, in patients with BCa, decreased expression of tumor-suppressive miRNAs such as miR-145 has been linked to advanced stages and a poor prognosis [80]. Furthermore, a miRNA profile associated with cuproptosis, a particular type of cell death, has been demonstrated to predict BCa prognosis and immunological landscape [95]. These findings highlight the great potential of miRNAs for enhancing the clinical management of BCa (Fig. 3B,C).

### 4.2 The Role of Long Non-Coding RNAs in Bladder Cancer Development

LncRNAs are highly dysregulated in BCa and play critical roles in carcinogenesis, progression, and prognosis [96]. As miRNAs, they regulate a variety of biological functions, including proliferation, cell cycle, apoptosis, migration, invasion, metabolism, and drug resistance [96], and their abnormal expression is frequently associated with patient prognosis and the appearance of metastases [88]. Specific lncRNAs, including UCA1, GAS5, and UNMIBC, have been shown to have predictive relevance in NMIBC [83]. A possible candidate therapeutic target is UCA1, which has been extensively studied and demonstrated to stimulate BCa cell growth and block apoptosis [83]. LncRNA MIR31HG and its splice variants control cell proliferation and migration, and they have prognostic significance in MIBC [84]. LncRNA SNHG1 has been found to stimulate BCa growth by increasing EZH2 protein expression and inhibiting KLF2 gene transcription [82]. Additionally, it has been reported that tumor-suppressive lncRNAs can be silenced by epigenetic changes, such as hypermethylation, which then promotes tumor growth [97], as the case of hypermethylated LINC00683 and MSC-AS1 that have been linked to poor overall survival in BCa patients [98]. Prognostic models based on metabolism-related lncRNAs have been developed to predict patient outcomes in BCa [99]. In parallel, several lncRNAs associated with neutrophil extracellular traps (NETs), including MAP3K4-AS1, MIR100HG, NKILA, and THY1-AS1, have been linked to prognosis and tumor progression [100]. NETs themselves, beyond their physiological role in pathogen capture, have been implicated in cancer advancement and metastasis, further supporting the relevance of NET-related lncRNAs as potential markers of disease behavior [101] (Fig. 3B,C). Their involvement in these pathways underscores the complex regulatory roles of lncRNAs and highlights their considerable potential as diagnostic, prognostic, and therapeutic targets in BCa.

### 4.3 Circular RNAs Contribution to Bladder Cancer Initiation and Progression

CircRNAs are a novel type of ncRNA that play important functions in physiological and developmental processes [89]. They can stimulate tumor growth through a variety of mechanisms, including interactions with miRNAs that influence transcription factors and both classical and nonclassical tumor signaling pathways [89]. More interestingly, they can function as miRNA sponges, effectively reducing miRNA action, or as protein baits or antagonists, then regulating protein function [89] (Fig. 3B,C). It is worth noting that the miRNA sponge process, in which circRNAs attach to and sequester miRNAs, de-represses the miRNA target genes and promotes or suppresses cancer phenotypes, based on target topology. CircRNAs are frequently dysregulated in BCa and have been linked to numerous clinicopathological features, prognosis, and chemosensitivity [89]. Because they persist longer in biofluids and are more resistant to exonuclease degradation than linear RNAs, they represent more reliable biomarkers [102,103] and have therefore emerged as promising candidates for BCa diagnosis and prognosis [89]. CircRNA MYLK, for example, has been found to accelerate BCa progression by altering the CCND3 level through a mechanism involving miR-34a [86]. In contrast, circRNA ACVR2A has been shown to inhibit BCa cell growth and metastasis via the miR-626/EYA4 axis [85]. Furthermore, the circ_100242/miR-145 pathway has been linked to the genesis and progression of BCa [104]. These findings, therefore, highlight the increasing role of circRNAs in BCa biology and their potential in the clinical management of BCa (Fig. 3B,C).

### 4.4 NcRNAs as Therapeutic Payloads for Nanometric Delivery

The established role of ncRNAs in driving and regulating BCa pathology indicates them as ideal molecular payloads for targeted therapeutic delivery via nanovectors, such as exosomes and nanorobots. The challenge in using ncRNAs therapeutically, namely their persistence in biological fluids and their poor delivery efficiency, is precisely what nanomedicine aims to solve. Candidate ncRNAs for nanovehicle delivery include, therefore, ncRNAs as diagnostic markers, tumor-suppressing ncRNAs, and inhibitors of oncogenic ncRNAs. More specifically, the restoration of miRNAs that are downregulated in cancer (tumor-suppressing miRNAs like miR-145) can result in the inhibition of tumor growth, and this observation makes them the prime candidates to load into exosomes for the subsequent nanorobot delivery. Additionally, therapeutic strategies often involve the delivery of small interfering RNAs (siRNAs) or antisense oligonucleotides (ASOs) designed to silence oncogenic ncRNAs (like UCA1 or oncogenic circRNAs). The protection offered by the exosome lipid bilayer, combined with the nanorobot active targeting, could be essential for maintaining the stability and efficacy of these highly sensitive molecules in the bladder environment. Finally, it has been abundantly reported that miRNAs and circRNAs are found in urine and blood [65] represent excellent biomarkers. When integrated with nanorobots designed for *in situ* capture and biosensing, these ncRNA signatures can increase the efficacy of LB diagnostics (Fig. 3D). Basically, the purpose of a nanorobot-exosome hybrid system is to shield these sensitive ncRNAs from degradation, enable their passage through biological barriers, and ensure their high-fidelity release into cancer cells, thereby delivering a precise therapeutic effect.

## 5 The Integrated Synergy: Nanorobots, Exosomes and ncRNAs for Hybrid Therapy

The convergence of nanorobots, exosomes and ncRNAs represents one of the most promising frontiers in oncological nanomedicine, as each technology addresses a key limitation of the others. A particularly compelling perspective is the development of a hybrid delivery strategy that leverages the long-range and active propulsion of nanorobots together with the loading and intracellular release capabilities of exosomes to achieve targeted and high-fidelity transport of ncRNA-based therapeutics [74] (Table 5).

**Table 5:** Nanorobot-exosome-ncRNA integration as proposed hybrid system.

Component	Function	Advantages
Nanorobot	Active propulsion and targeting	Overcomes GAG barrier and urinary clearance
Exosome	Natural carrier for ncRNA	High biocompatibility, stable cargo protection
ncRNA	Precision therapeutic payload	Targets oncogenic pathways

Abb: ncRNA, non-coding RNA; GAG, glycosaminoglycan.

**Figure 4:**
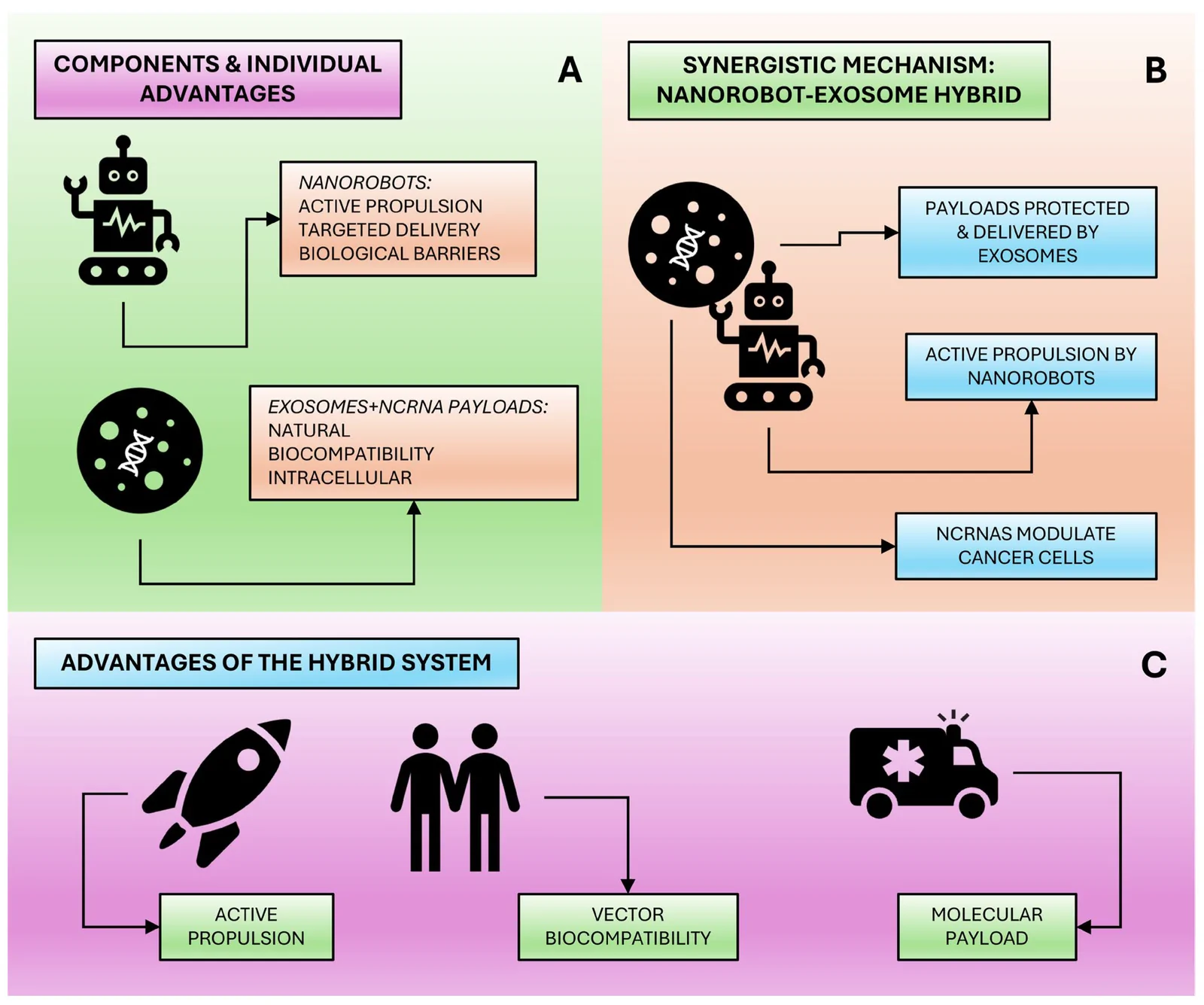
**Synergistic mechanism of the hybrid nanorobot-exosome system.** (**A**) Nanorobots and exosomes are characterized by peculiar molecular features that make them translationally unique; (**B**) The proposed synergistic mechanism integrates the active functionality of nanorobots with the biocompatible properties of exosomes; (**C**) Exosomes act as natural carriers, protecting and transporting non-coding RNA (ncRNA) cargo, while nanorobots provide active propulsion, overcoming the passive nature of exosomes: this combination enhances targeting precision and delivery efficiency at the tumor site. The figure is original and was generated using Microsoft PowerPoint (Version 16.107), utilizing the native vector-based iconography provided within the software. NCRNA, non-coding RNA.

While no studies have yet reported the use of nanorobots to deliver exosomes in BCa, the combined use of these two platforms offers a compelling conceptual path forward. By combining the precise mobility and targeting abilities of nanorobots with the intrinsic biocompatibility and signaling capacity of exosomes, this strategy provides a coherent framework for advancing localized therapies. Employing exosomes loaded with selected ncRNAs together with nanorobot-guided targeting could yield an effective method for delivering therapeutic agents directly to BCa (Fig. 4A).

It is crucial to clarify that the proposed exosome–nanorobot hybrid remains a forward-looking design concept and it should not be misinterpreted as a platform that has already achieved for *in vivo* validation for bladder cancer treatment [29,30]. While several interesting studies have successfully validated urease-powered nanorobots as active carriers, or demonstrated that engineered exosome-derived vesicles can function as motile delivery systems, an integrated assembly that merges both functionalities into a single and unified construct has yet to be realized [29,30]. However, to test this hybrid model, here we propose specific and measurable design criteria, accounting for parameters that mimic conditions of the bladder environment, including urine dilution, voiding cycles and the resistance to natural tissue barriers [30,44]:

- *Attachment chemistry (stability vs. function).* To overcome urinary washout and ensure clinical efficacy, the bond between the exosome and the motile unit must maintain stability for several hours without hindering mobility or biological interaction, and performance must align with established *in vivo* retention benchmarks [29,30]. Validation requires rigorous testing in urine-simulating media (accounting also for pH fluctuations) to confirm colloidal stability, size distribution and preservation of biological identity and cell-internalization ability [30,44].
- *Release triggers (spatiotemporal control).* To achieve precise spatiotemporal control, the release mechanism (whether ultrasound-mediated, pH-sensitive or catalytic) must be clearly defined and validated. Outcome is measured by intramucosal penetration depth, tumor accumulation and the effective activation of the payload, under simulated intravesical conditions [29,30,44]. Ultrasound-mediated activation serves as a proven benchmark, demonstrating that controlled triggers directly translate to better *in vivo* antitumor performance, providing a robust framework for evaluating future hybrid designs [30].
- *Safety thresholds (local tolerability).* To ensure local tolerability, safety must be correlated to quantitative intravesical markers, specifically maintaining mucosal integrity and controlling urinary pH fluctuations. Since catalytic motors can produce by-products that alter the local environment or irritate tissue, monitoring these parameters is essential [30,44]. In this case, critical benchmarks are represented by urease-driven systems: even they can alkalinize urine, evidence confirms that *in vivo* this remains within a tolerated physiological range. These data should be used to establish stringent dose limits and explicit stopping-rules for future hybrid designs, ensuring that therapeutic activity does not compromise patient safety [30].

Therefore, any hybrid architecture must demonstrate that its added complexity provides a clear advantage over simpler alternatives. This must be proven through key metrics, such as retention after voiding, improved transmucosal transport or deeper intratumoral penetration, which currently distinguish active delivery systems from passive carriers [29,30,44].

### 5.1 Nanorobots as Active Vectors for Therapeutic Exosomes

Exosomal delivery is mainly constrained by passive diffusion and rapid urinary clearance. While recent hydrogel-based systems, such as the magnetic chitosan-nanoparticle platform described by Zheng and colleagues, effectively prolong retention and structural integrity, they remain inherently passive. These scaffolds successfully anchor vesicles within the bladder wall and facilitate sustained release of regenerative factors like VEGF and NGF, thereby restoring contractility and promoting neurotrophic repair [105]. Even with enhanced anchoring, such platforms lack active tissue-penetration capabilities. Integrating these bioengineered composites with nanorobotic propulsion offers a powerful strategy, combining the localized retention achieved with hydrogels and the autonomous movement needed to improve ncRNA delivery in the confined bladder environment. Building on this concept, nanorobots driven by chemical or magnetic propulsion could transport exosome-loaded ncRNAs, such as miR-143, miR-203, or inhibitors of oncogenic lncRNAs, ensuring precise and focal accumulation within neoplastic tissue.

This hybrid approach would provide (a) active propulsion, since nanorobots overcome urinary clearance and penetrate the bladder mucus, (b) vector biocompatibility, since exosomes offer a natural delivery platform, reducing immunogenicity and ensuring better efficiency of intracellular ncRNA transfer and (c) molecular payload, since ncRNAs (designed to silence oncogenic genes) provide the precision therapeutic action (Fig. 4B). To provide a more tangible representation of the hybrid nanorobot-exosome platform, we envision a design in which actively propelled nanorobots serve as the outer navigational scaffolds, while engineered exosomes are reversibly anchored to their surfaces through ligand/receptor or magnetic tagging interfaces. In this configuration, nanorobots operate as directional carriers able to cross the urinary environment and breach the GAG layer, whereas the attached exosomes preserve their biological integrity and encapsulate ncRNA cargo until reaching the tumor niche. Once localized at the target site, environmental cues such as pH, enzymatic activity, or externally applied magnetic stimuli could trigger the controlled release of the exosomes [18], enabling efficient uptake by cancer cells and precise intracellular delivery of the therapeutic ncRNAs. This conceptual arrangement highlights the division of roles within the system and offers a plausible architectural project for future implementation. This combination represents an engineering solution that mixes the kinetic functionality of the nanorobots with the advanced biological communication capability of the exosome. The exact delivery of ncRNAs directly into tumor cells, while reducing off-target effects on healthy tissues, is made possible by nanorobots, which provide previously spatial and temporal control over drug delivery [19]. Therapeutic ncRNAs can be delivered directly to BCa cells via nanorobots, which can carry a variety of payloads, such as genes and sensing molecules [35] (Fig. 4C).

Strategies based on nanomaterials, which are technologically comparable to nanorobotics, are being developed to target lncRNAs in cancer cells through the use of Clustered Regularly Interspaced Short Palindromic Repeats (CRISPR)-based gene editing tools, RNA interference (RNAi) technologies, and ASOs [23,24]. These potent molecular tools provide extremely selective therapeutic interventions by precisely modulating the expression of tumor-suppressive or oncogenic lncRNAs [23,24]. It may be possible to create smart drug delivery systems that can sense the tumor microenvironment and release therapeutic ncRNAs just when and where they are required by combining such tools with nanorobots. By increasing drug concentration at the tumor site and reducing systemic exposure, local drug delivery systems that make use of nanotechnology have demonstrated the potential to improve the effectiveness of BCa treatments [45]. Additionally, BCa models have effectively used exosome-mediated transport of artificial circRNAs for gene therapy, demonstrating the feasibility of utilizing nanoscale carriers for ncRNA delivery [62]. These developments imply that, in the treatment of BCa, nanorobots may provide an even more accurate and focused delivery method for different therapeutic ncRNAs (Fig. 4C).

### 5.2 Advanced Diagnostic Assays and Exosome/ncRNA Scavengers

Beyond therapy, synergy between these three different molecular systems can fundamentally optimize diagnosis and intervention against oncogenic signaling in BCa. A first area of application is the use of nanorobots to enhance LB. Nanorobots equipped with specific aptamers or antibodies could navigate the bladder lumen and capture circulating tumor-derived exosomes *in situ*, many of which carry oncogenic ncRNAs. Collecting these vesicles directly at the tumor interface, rather than from diluted urine samples, would strongly improve the sensitivity and specificity of ncRNA-based LB diagnostics for BCa. In this perspective, exosomes are essential for LB applications because of their distinct cargo, which reflects the physiological and pathological conditions of the parental cells that released them [49]. NcRNAs, which are abundant in exosomes, are important for the development of tumors and can be useful biomarkers for the diagnosis and prognosis of BCa [20,65]. The identification of particular oncogenic ncRNAs carried by exosomes, such as exosome-derived circSCL38A1, which promotes a malignant phenotype [58] or lncRNA BCYRN1, which has demonstrated promise as a therapeutic target and diagnostic marker in serum exosomes of BCa patients [66], holds great clinical potential.

A second relevant perspective is the potential use of nanorobots as scavengers and inhibitors of tumor-derived exosomes. By directly targeting these vesicles, nanorobots could interfere with the communication and signaling pathways that support BCa progression. This may include delivering therapeutic agents to the exosomes, blocking their release, or preventing their uptake by recipient cells. To further reduce their pro-tumorigenic activity, nanorobots could be engineered to carry compounds capable of disrupting exosomal membranes or inhibitors of exosome biogenesis pathways, such as those acting on the ESCRT machinery [73,74]. Alternatively, nanorobots may function as exosome scavengers, physically entrapping and removing tumor-derived exosomes from circulation to mitigate their roles in immunosuppression and metastasis [48,74]. This dual approach, simultaneously delivering therapeutic agents and modulating exosome activity, offers a promising direction for enhancing the effectiveness of BCa treatment.

## 6 Novel Challenges and Future Plans

There is considerable potential for continued research in this field, with many important directions still to be explored. Although nanorobots, exosomes, and ncRNAs each offer remarkable opportunities, their full clinical integration remains complex. Future work will need to address not only the properties of these individual components but also the engineering, standardization, and large-scale production required to develop a reliable nanorobot-exosome hybrid system.

### 6.1 Future Research Avenues

**Figure 5:**
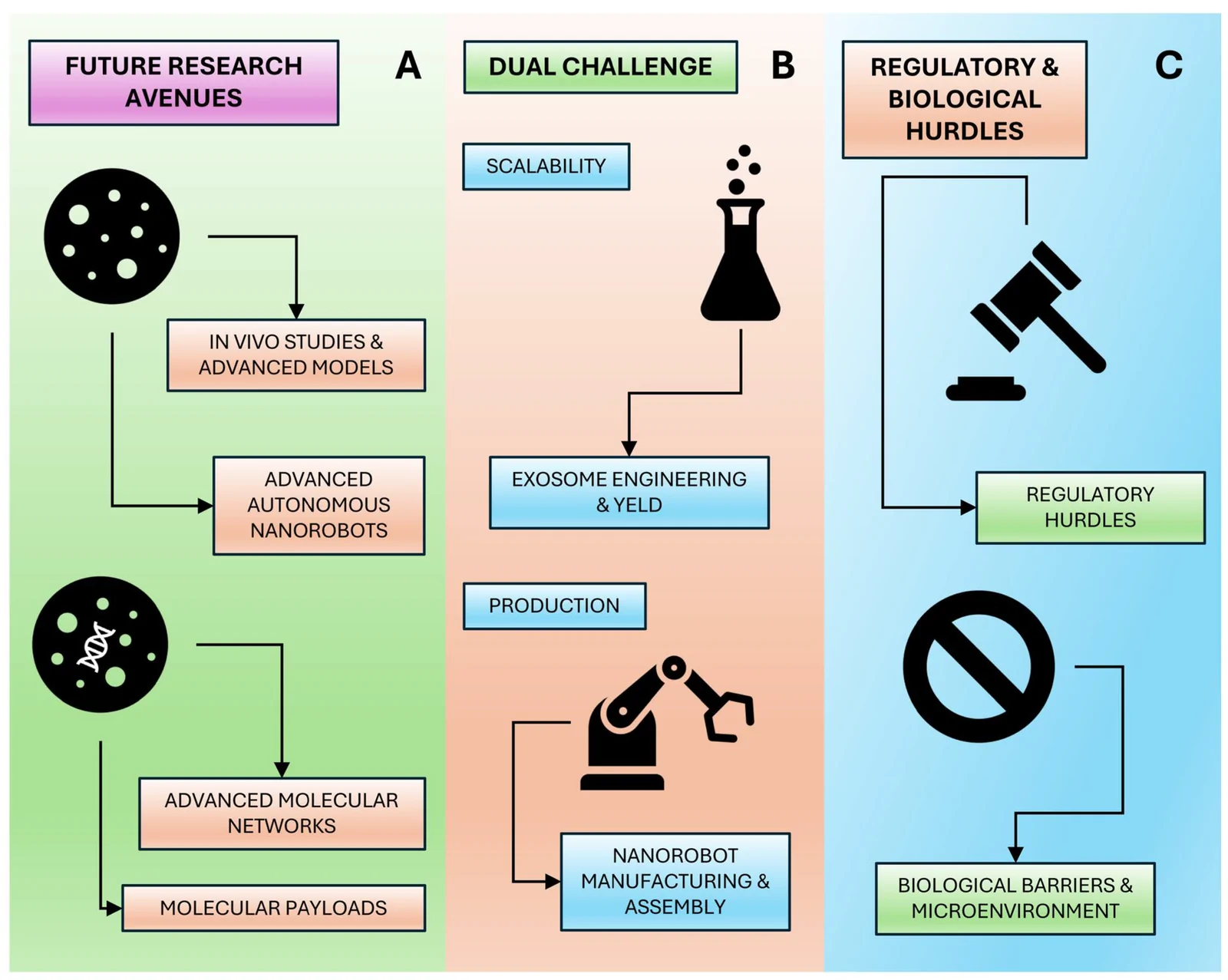
**Future research directions and challenges.** (**A**) Future research will focus on *in vivo* studies and advanced models, with the final goal to design advanced and autonomous nanorobots optimized for the delivery of therapeutic non-coding RNA (ncRNA) cargo; (**B**) Future challenges are represented by engineering and optimizing exosome production and the manufacturing and assembly of nanorobots; (**C**) Key obstacles include the need to overcome biological barriers and the need to establish a regulatory framework consistent with the use of such a hybrid system. The figure is original and was generated using Microsoft PowerPoint (Version 16.107), utilizing the native vector-based iconography provided within the software.

*In vivo* studies are urgently required to clearly demonstrate the kinetic and therapeutic advantage of active delivery via nanorobots compared to passive exosomal administration in the animal model of BCa. Accordingly, more preclinical research is needed in more intricate *in vivo* models that better mimic the human condition [29]. These models, which include organoids and patient-derived xenografts (PDX) [106] can better capture the intricacy of BCa in humans and offer more interpretable data. By investigating the synergistic effects of combining ncRNA treatments, exosomes and nanorobots in these advanced systems, there is great potential to achieve more persistent and effective responses (Fig. 5A).

To increase their accuracy and effectiveness, more advanced autonomous nanorobots with improved targeting capabilities, possibly directed by biosensors or real-time imaging, must be developed [18]. Real-time feedback on drug release and nanorobot localization could be obtained through integration with advanced imaging methods like optical coherence tomography or photoacoustic imaging, allowing for dynamic therapy changes [43].

When compared to other advanced intravesical delivery strategies currently under investigation, such as mucoadhesive hydrogels, thermosensitive polymeric depots, and passive nanoparticle formulations, the proposed nanorobot-exosome hybrid system exhibits a distinct functional profile. Hydrogels and polymeric systems improve dwell time, but remain fully diffusion dependent, while passive nanoparticles offer enhanced stability yet still succumb to urinary washout and limited penetration through the GAG layer [14]. In contrast, the hybrid platform combines the biological compatibility and molecular specificity of exosomal ncRNA delivery with the active propulsion of nanorobots, enabling guided motion, sustained bladder-wall engagement, and targeted release at tumor sites [18]. This comparison underscores both the promise of the hybrid approach and the engineering challenges that accompany its complexity, particularly in terms of scalable fabrication and coordinated control of its integrated components.

The translational potential of the nanorobot-exosome-ncRNA platform centers on the clinical feasibility of intravesical instillation as the primary route of administration. This route inherently exposes the system to challenges such as urine flow-induced dilution and washout, which further justify the need for active propulsion to maintain contact with the bladder wall and sustain targeted delivery [11]. At the same time, intravesical administration offers advantages over systemic delivery, including reduced systemic exposure, lower toxicity, and direct access to the tumor-bearing mucosa, thereby avoiding the biodistribution barriers and off-target sequestration typical of intravenous delivery [45]. Emphasizing this administration strategy highlights both the clinical practicality of the approach and the specific engineering features that render the hybrid system suitable for localized BCa therapy.

### 6.2 The Dual Challenge of Scalability and Production

The establishment of scalable and affordable production techniques will also be necessary for the actual application of these cutting-edge treatments [31]. This is challenging for the hybrid system, as it requires scaling two complex components simultaneously. The clinical scalability of exosome and ncRNA platforms is restricted not only by the challenge of yield, but also by significant discrepancies across studies regarding pre-analytical steps, separation techniques, characterization methods and cargo-loading assessments, all of which hinder their large-scale clinical application [107]. Consequently, establishing consensus-based reporting frameworks has become a fundamental requirement for effective clinical translation [108,109].

The Minimal Information for Studies of Extracellular Vesicles (MISEV) 2023 guidelines offer a field-wide consensus structure for EV research, addressing nomenclature, pre-analytical variables, various separation and concentration strategies, storage protocols and requirements for purpose-adequate characterization. This framework serves as the reference standard when evaluating and developing scalable workflows [108]. EV-TRACK consortium further supports these guidelines by turning methodological transparency into a practical process through its curated EV-METRIC checklist. This tool takes in consideration critical experimental parameters (from sample type and pre-analytics to isolation and characterization), thereby overcoming the reproducibility barriers that often prevent clinical improvement [109]. In particular, standardized EV isolation has to be treated as a well definite sequence where source variables, processing timelines and storage conditions are primary determinants of yield and molecular profile, rather than minor procedural details [108]. Selecting an isolation method (such as SEC, filtration or affinity capture) involves a careful balance between recovery and purity and, consequently, detailed reporting is mandatory to ensure reproducibility across different laboratories [108]. Furthermore, since non-vesicular extracellular particles (NVEPs) and co-isolates can significantly interfere with results, clinical pipelines must discard the assumption of pure exosomes in favor of transparent and rigorous characterization of the final product [108]. As far as ncRNA loading in concerned, modern standardization efforts provide practical reporting frameworks designed to differentiate between endogenous and exogenous loading. These guidelines require thorough purification and experimental controls to ensure that free or aggregated nucleic acids are not mistaken for loaded EV cargo [110]. In this regard, Roerig and Schulz-Siegmund propose a testable framework for EV-cargo reporting by emphasizing the evaluation of critical variables, such as the EV/cargo ratio, buffer composition, energy input and isolation strategy, and the quantification of loading efficiency through standardized metrics, including encapsulation efficiency, loading capacity and mass balance, to ensure reproducibility and identify loss patterns in large-scale production [110]. Aligning these processes with MISEV2023 and EV-TRACK guidelines facilitates clinical translation by transforming technical challenges into a structured checklist, thereby ensuring that exosome-based therapies are both reproducible and adaptable to rigorous good manufacturing practice (GMP)-compliant workflows [108,109,110].

Similar challenges arise in nanorobot manufacturing and functionalization, where producing complex devices with consistent performance at scale continues to pose substantial engineering obstacles [19]. The most critical issue is the development of reliable and scalable methods for stably attaching engineered exosomes to the nanorobot surface while preserving their biological activity. Standardizing these procedures will be essential to ensure the reproducibility and clinical applicability of such combined systems (Fig. 5B). An additional layer of complexity arises from the choice of exosome source, which directly influences production scalability, therapeutic consistency, and the regulatory pathway of the hybrid system. Exosomes derived from patient-specific cells may offer superior biocompatibility, but pose substantial hurdles in large-scale manufacturing, whereas standardized producer lines improve yield yet raise questions about functional equivalence and long term safety [107].

### 6.3 Regulatory and Biological Hurdles for Integrated Therapies

The regulatory landscape for such integrated approaches represents a major challenge. The guidelines issued by the Food and Drug Administration (FDA) and European Medicines Agency (EMA) for combined products that combine an active device (nanorobot) with a gene-modulating agent (ncRNA) and a biological component (exosome) remain complex and still evolving. Regulatory bodies worldwide are continuing to refine frameworks for approving gene-based therapies and advanced nanomedicines, and the distinctive features of nanorobot-exosome-ncRNA constructs will inevitably demand tailored assessment and dedicated regulatory pathways (Fig. 5C). From a regulatory perspective, hybrid platforms that integrate micro- or nano-devices with biologically derived vesicles and nucleic acid therapeutics pose significant classification challenges. Existing guidance for nanomedicines and Advanced Therapy Medicinal Products (ATMPs) offers only partial direction, as the convergence of device-driven propulsion, biological carriers, and gene-modulating payloads aligns this technology with emerging categories of combination products [19,74,91]. Experience from nanoparticle-based gene therapies and exosome-derived biologics further indicates that issues such as manufacturing consistency, biodistribution control, and long-term safety testing will be central to future regulatory evaluation [46]. Considering these factors helps position the proposed hybrid system within current approval pathways and highlights the translational hurdles that must still be addressed.

Furthermore, carefully planned clinical trials are required to assess the safety and effectiveness of these integrated therapies in patients with BCa. As for safety, the engineering steps required to functionalize exosomes, particularly the conjugation of nanorobot anchoring moieties or targeting ligands, could introduce new immunogenic epitopes, necessitating careful evaluation of host immune activation [46]. Besides, the inorganic/organic nature of the hybrid constructs requires rigorous studies on long term biodistribution, degradation kinetics, and clearance, together with assessments of potential off-target effects arising from excessive exosome load or unintended accumulation of nanorobot remnants. Addressing these aspects will be essential for translating the platform into a clinically acceptable and regulatory-compliant therapeutic strategy.

It is important to note that several biological barriers must be addressed to ensure effective delivery of these therapeutics to BCa [18]. One key challenge is the GAG layer lining the bladder wall, which can hinder the penetration of intravesically administered agents: this obstacle may be partially mitigated by the active propulsion of nanorobots [44]. However, treatment efficacy is also shaped by the tumor microenvironment, whose immunosuppressive features and altered extracellular matrix can limit the performance of both nanorobot- and exosome-based approaches [56] (Fig. 5C). Further progress will depend on a deeper understanding of the specific ncRNA cargo carried by exosomes and the mechanisms through which these molecules influence tumor initiation and progression [48]. Clarifying how exosomal ncRNAs regulate oncogenic pathways will be essential for designing more precise and effective therapeutic payloads.

## 7 Conclusion: New Directions in Bladder Cancer Treatment

BCa therapy is evolving rapidly, moving beyond conventional, passive and diffusion-limited approaches to enter a period of advanced and actively-guided nanomedicine. The primary limitation of traditional intravesical therapy lies in overcoming physiological barriers, specifically the fast urinary clearance and the protective GAG layer, to achieve effective drug concentration at the tumor site. The convergence of advanced technologies, such as nanotechnology, exosome biology and ncRNA studies, defines a new therapeutic paradigm that promises enhanced accuracy and effectiveness. This review highlights the synergistic potential of three complementary components which, when integrated, give rise to a unified and powerful hybrid system. In summary:

- *Nanorobots* contribute the capacity for active targeting, providing the kinetic drive necessary to overcome biological barriers and urine flow, and enabling precise accumulation at the tumor site. Their ability to deliver therapeutic payloads with high spatial and temporal control offers a means to reduce off-target effects and enhance overall treatment efficiency [19];
- *Exosomes* add an equally critical dimension, functioning as natural biocompatible carriers with low immunogenicity and an inherent ability to protect and transfer sensitive molecular cargo. Their established role in BCa biology also makes them valuable as non-invasive biomarkers for diagnosis and prognosis, while their natural affinity for specific cell types and their efficiency in transporting ncRNAs provide clear advantages over synthetic nanoparticles [26];
- *NcRNAs* supply the molecular precision required for targeted intervention. As key regulators of pathways involved in BCa initiation and progression, tumor-suppressive miRNAs and inhibitors of oncogenic lncRNAs offer highly specific therapeutic opportunities. Their integration into an exosome-nanorobot platform underscores the potential of this hybrid approach to achieve a refined and mechanism-based modulation of oncogenic signaling.

This nanorobot-exosome hybrid system represents an integrated engineering strategy that combines the kinetic and targeting capabilities of nanotechnology with the sophisticated biological communication inherent to natural vesicles. Together, these complementary elements offer a promising foundation for more individualized, effective, and less toxic approaches to BCa therapy. The successful translation of this concept from laboratory development to clinical application will depend on overcoming several key challenges. Addressing biological barriers and rapid urine clearance will require improved nanorobot and exosome-based carrier designs capable of achieving reliable accumulation and retention at the tumor site [44]. Equally important are rigorous preclinical evaluations to establish safety, along with scalable and cost-efficient manufacturing systems to support reproducible production. Clear regulatory pathways will also be essential to facilitate approval and broaden clinical adoption. Finally, the convergence of these technologies holds the potential to strongly enhance diagnostic precision, therapeutic performance and overall patient outcomes, representing a significant step forward in the treatment of BCa.

## Data Availability

Not applicable.

## Abbreviations

ADC	Antibody-drug conjugates
ASO	Antisense oligonucleotide
ATMP	Advanced Therapy Medicinal Product
BCG	Bacillus Calmette-Guérin
BCa	Bladder cancer
BMSC	Bone marrow mesenchymal stem cells
circRNA	Circular RNA
CIS	Carcinoma *in situ*
CRISPR	Clustered Regularly Interspaced Short Palindromic Repeats
ctDNA	Circulating tumor DNA
D_t_	Diffusion Coefficient
EMA	European Medicines Agency
EMT	Epithelial-mesenchymal transition
ESCRT	Endosomal Sorting Complex Required for Transport
EV	Extracellular vesicle
FDA	Food and Drug Administration
GAG	Glycosaminoglycan
GMP	Good manufacturing practice
GSH	Glutathione
HAL	Hexaminolevulinate
LB	Liquid biopsy
lncRNA	Long non-coding RNA
MIBC	Muscle-invasive bladder cancer
mRNA	Messenger RNA
miRNA	MicroRNA
MISEV	Minimal Information for Studies of Extracellular Vesicles
MNR	Micro- and nano-robot
MOF	Metal-organic framework
MRI	Magnetic Resonance Imaging
ncRNA	Non-coding RNA
NET	Neutrophil extracellular trap
NMIBC	Non-muscle-invasive bladder cancer
NVEP	Non-vesicular extracellular particles
PDX	Patient-derived xenograft
PET	Positron Emission Tomography
RNAi	RNA interference
SEC	Size exclusion chromatography
siRNA	Small interfering RNA
TAM	Tumor-associated macrophage
TMB	Tumor mutation burden
TURBT	Transurethral resection of bladder tumor
VI-RADS	Vesical Imaging-Reporting and Data System
WHO	World Health Organization
